# Aerial Mapping of Forests Affected by Pathogens Using UAVs, Hyperspectral Sensors, and Artificial Intelligence

**DOI:** 10.3390/s18040944

**Published:** 2018-03-22

**Authors:** Juan Sandino, Geoff Pegg, Felipe Gonzalez, Grant Smith

**Affiliations:** 1 Insitute for Future Environments; Robotics and Autonomous Systems, Queensland University of Technology (QUT), 2 George St, Brisbane City, QLD 4000, Australia; felipe.gonzalez@qut.edu.au; 2 Horticulture & Forestry Science, Department of Agriculture & Fisheries, Ecosciences Precinct, 41 Boggo Rd Dutton Park, QLD 4102, Australia; geoff.pegg@daf.qld.gov.au; 3 BioProtection Technologies, The New Zealand Institute for Plant & Food Research Limited, Gerald St, Lincoln 7608, New Zealand; grant.smith@plantandfood.co.nz

**Keywords:** *Austropuccinia psidii*, drones, hyperspectral camera, machine learning, *Melaleuca quinquenervia*, myrtle rust, non-invasive assessment, paperbark, unmanned aerial vehicles (UAV), xgboost

## Abstract

The environmental and economic impacts of exotic fungal species on natural and plantation forests have been historically catastrophic. Recorded surveillance and control actions are challenging because they are costly, time-consuming, and hazardous in remote areas. Prolonged periods of testing and observation of site-based tests have limitations in verifying the rapid proliferation of exotic pathogens and deterioration rates in hosts. Recent remote sensing approaches have offered fast, broad-scale, and affordable surveys as well as additional indicators that can complement on-ground tests. This paper proposes a framework that consolidates site-based insights and remote sensing capabilities to detect and segment deteriorations by fungal pathogens in natural and plantation forests. This approach is illustrated with an experimentation case of myrtle rust (*Austropuccinia psidii*) on paperbark tea trees (*Melaleuca quinquenervia*) in New South Wales (NSW), Australia. The method integrates unmanned aerial vehicles (UAVs), hyperspectral image sensors, and data processing algorithms using machine learning. Imagery is acquired using a Headwall Nano-Hyperspec${}^{®}$ camera, orthorectified in Headwall SpectralView${}^{®}$, and processed in Python programming language using eXtreme Gradient Boosting (XGBoost), Geospatial Data Abstraction Library (GDAL), and Scikit-learn third-party libraries. In total, 11,385 samples were extracted and labelled into five classes: two classes for deterioration status and three classes for background objects. Insights reveal individual detection rates of 95% for healthy trees, 97% for deteriorated trees, and a global multiclass detection rate of 97%. The methodology is versatile to be applied to additional datasets taken with different image sensors, and the processing of large datasets with freeware tools.

## 1. Introduction

Exotic pathogens have caused irreversible damage to flora and fauna within a range of ecosystems worldwide. Popular outbreaks include the enormous devastations of chestnut blight (*Endothia parasitica*) on American chestnut trees (*Castanea dentata*) in the U.S. [1,2,3], sudden oak death (*Phytophthora ramorum*) on oak populations (*Quercus agrifolia*) in Europe, California, and Oregon [4,5,6], dieback (*Phytophthora cinnamomi*) on hundreds of hosts globally [7,8,9], and myrtle rust (*Austropuccinia psidii*) on Myrtaceae family plants in Australia [10,11,12,13]. The effects of the latter case have raised national alerts and response programmes given the extensive host range and the ecological and economic importance of Myrtaceae plants in the Australian environment [14,15,16,17]. As a result, various surveillance and eradication programmes have been applied in an attempt to minimise the impacts invasive pathogens cause on local hosts such as dieback in the Western Australia Jarrah forests [18], sudden oak death in the tan oak forests of the U.S. [19], and rapid ohia death (*Ceratocystis fimbriata*) on ohia trees (*Metrosideros polymorpha*) in Hawaii [20].

Modern surveillance methods to map hosts vulnerable to and affected by exotic pathogens can be classified in site-based and remote sensing methods, according to Lawley et al. [21]. Site-based approaches are commonly small regions used to collect exhaustive compositional and structural indicators of vegetation condition with a strong focus on biophysical attributes of single vegetation communities [22,23]. These methods, nonetheless, require deep expertise and time to conduct experimentation, data collection, and validation that, along with their limited area they can cover, represent a challenge while assessing effects on a broad scale [24]. Research has also suggested the design of decision frameworks to monitor and control the most threatened species [17,25,26]. Although these models can determine flora species that require immediate management control, limitations on the amount of tangible, feasible, and broad quantified data of vulnerable host areas [21] have resulted in lack of support from state and federal governments [11].

The role of remote sensing methods to assess and quantify the impacts of invasive pathogens in broad scale has increased exponentially [27,28]. Standard approaches comprise the use of spectral and image sensors through satellite, manned, and unmanned aircraft technology [29]. Concerning sensing technology by itself, applied methods by research communities include the use of non-imaging spectroradiometers, fluorescence, multispectral, hyperspectral, and thermal cameras, and light detection and ranging (LiDAR) technology [30,31,32,33]. These equipment are usually employed for the calculation of spectral indexes [34,35,36,37] and regression models in the host range [38,39]. Nevertheless, these methods are mainly focused on quantification and distribution, among other physical properties of flora species.

Satellite and manned aircraft surveys have reported limitations concerning resolution, operational costs, and unfavourable climate conditions (e.g., cloudiness and hazard winds) [40]. In contrast, the continuous development of unmanned aerial vehicles (UAVs) designs, navigation systems, portable image sensors and cutting-edge machine learning methods allow unobtrusive, accurate, and versatile surveillance tools in precision agriculture and biosecurity [41,42,43,44]. Many studies have positioned UAVs for the collection of aerial imagery in applications such as weed, disease, and pest mapping and wildlife monitoring [21,45,46,47]. More recently, unmanned aerial systems (UASs) have been deployed in cluttered and global positioning system (GPS)-denied environments [48].

Approaches to the use of UAVs, hyperspectral imagery and artificial intelligence are gaining popularity. For example, Aasen et al. [49] deployed UAS to boost vegetation monitoring efforts using hyperspectral three-dimensional (3D) imagery. The authors of Nasi et al. [50] developed techniques to assess pest damages at canopy levels using spectral indexes and k-nearest neighbour (k-NN) classifiers, achieving global detection rates of 90%. Similar research focused on disease monitoring, however, has been limited. The authors of Calderon et al. [51], for instance, evaluated the early detection and quantification of verticillium wilt in olive plants using support vector machines (SVMs) and linear discriminant analysis (LDA), obtaining mixed accuracy results among the evaluated classes of infection severity (59–75%) [52]. The authors of Albetis et al. [53] presented a system to discriminate asymptomatic and symptomatic red and white vineyard cultivars by *Flavescence doree*, using UAVs, multispectral imagery, and up to 20 data features, collecting contrasting results between the cultivars and maximum accuracy rates of 88%. In sum, the integration of site-based and remote sensing frameworks have boosted the capabilities of these surveillance solutions by combining data from abiotic and biotic factors and spectral responses, respectively [54]. However, this synthesis is still challenging due to the high number of singularities, data correlation, and validation procedures presented in each case study.

Considering the importance of site-based and remote sensing methods to obtain reliable and broader assessments of forest health, specifically, for pest and fungal assessments [55], this paper presents an integrated system that classifies and maps natural and plantation forests exposed and deteriorated by fungal pathogens using UAVs, hyperspectral sensors, and artificial intelligence. The framework is exemplified by a case study of myrtle rust on paperbark tea trees (*Melaleuca quinquenervia*) in a swamp ecosystem of Northeast New South Wales (NSW), Australia.

## 2. System Framework

**Figure 1:**
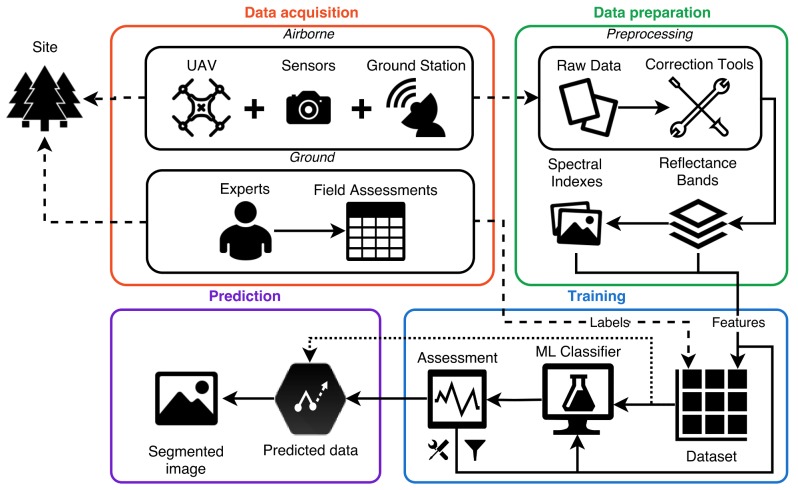
Pipeline process for the detection and mapping of alterations in natural and plantation forests by fungal diseases.

A novel framework was designed for the assessment of natural and plantation forests exposed and potentially exposed to pathogens as presented in Figure 1. It comprises four sections linked to each other, denoted as Data Acquisition, Data Preparation, Training, and Prediction. The system interacts directly and indirectly with the surveyed area to acquire information, preprocess and arrange obtained data into features, fit a supervised machine learning classifier, tune the accuracy and performance indicators to process vast amounts of data, and provide prediction reports through segmented images.

### 2.1. Data Acquisition

The data acquisition process involves an indirect data collection campaign using an airborne system, and direct ground assessments of the studied pathogen through exclusion trials, which are controlled by biosecurity experts. Airborne data is compiled using a UAV, image sensors, and a ground workstation in the site to acquire data above the tree canopy. Similarly, ground data is collected through field assessment insights by biosecurity experts. Field assessments bring several factors such as growth, reproduction, and regeneration on coppiced trees exposed to any specific pathogen. A database is created by labelling, georeferencing, and correlating relevant insights into every tested plant. Details on the studied area, flight campaigns, and field assessments can be found in Section 3.1, Section 3.2 and Section 3.3.

#### 2.1.1. UAV and Ground Station

This methodology incorporates a hexa-rotor DJI S800 EVO (DJI, Guangdong, China) UAV. The drone features high-performance brushless rotors, a total load capacity of 3.9 kg, and dimensions of 118 cm × 100 cm × 50 cm. It follows an automatic mission route using the DJI Ground Station 4.0 software, controlling the route, speed, height above the ground, and overlapping values remotely. It is worth mentioning, however, that other UAVs with similar characteristics can also be used and included into the airborne data collection process of Figure 1.

#### 2.1.2. Sensors

**Figure 2:**
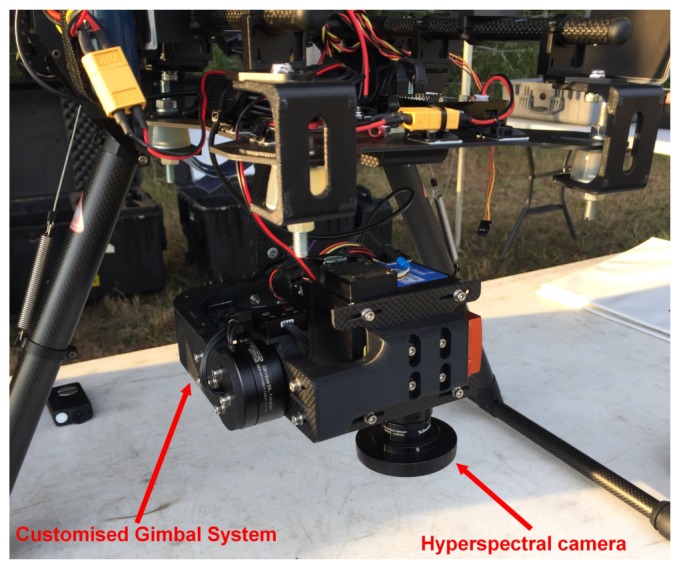
Assembly of the gimbal system and the Headwall Nano-Hyperspec${}^{®}$ camera into the DJI S800 EVO unmanned aerial vehicle (UAV).

Spectral data is collected using a Headwall Nano-Hyperspec${}^{®}$ hyperspectral camera (Headwall Photonics Inc., Bolton, MA, USA). This visible near infrared (VNIR) sensor provides spectral wavelength responses up to 274 bands, a wavelength range from 385 to 1000 nm, a spectral resolution of 2.2 nm, a frame rate of 300 Hz, spatial bands of 640 pixels, and a total storage limit of 480 GB. The camera is mounted in a customised gimbal system that augments data quality by minimising external disturbances such as roll, pitch, and yaw oscillations, as depicted in Figure 2.

### 2.2. Data Preparation

**Figure 3:**
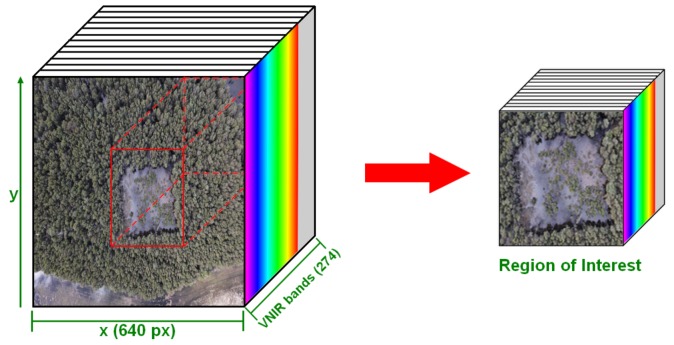
Creation of regions of interest in hyperspectral cubes.

Imagery is downloaded and fed into a range of software solutions to transform raw data into filtered and orthorectified spectral bands in reflectance. Using Headwall SpectralView${}^{®}$ software, raw hypercubes from the surveyed area were automatically processed. The preprocessing operations included radiance, orthorectification (through ground control points), and the white reference illumination spectrum (WRIS). The WRIS spectrum comes from the extraction of the radiance signature of a Spectralon target from an acquired image in the surveyed area. Using the orthorectified imagery and the WRIS, reflectance data and scene shading are calculated through CSIRO|Data61 Scyven 1.3.0. software [56,57]. Considering the massive amount of data contained for any orthorectified hyperspectral cube (4–12 GB), each cube is cropped to process regions of interest only (0.5–1 GB) and handle system resources efficiently, as shown in Figure 3.

An additional image that contains the localisation of tested trees in the exclusion trial is created. From a recovered red–green–blue colour model (RGB) image of the cropped hypercube, each tree is graphically labelled using their tracked GPS coordinates in Argis ArcMap 10.4. To handle all the data processing from the proposed system framework, Algorithm 1 was developed. These tasks were conducted with Python 2.7.14 programming language and several third-party libraries for data manipulation and machine learning, including Geospatial Data Abstraction Library (GDAL) 2.2.2 [58], eXtreme Gradient Boosting (XGBoost) 0.6 [59], Scikit-learn 0.19.1 [60], OpenCV 3.3.0 [61], and Matplotlib 2.1.0 [62].

**Algorithm 1** Detection and mapping of vegetation alterations using spectral imagery and sets of features.
*Required:* orthorectified layers (bands) in reflectance *I*. Labelled regions from field assessments *L*.
***Data Preparation***1:Load *I* data.2:S← Spectral indexes array from *I*.3:X← Features array [*I*, *S*].
***Training***4:Y← Labels array from dataset *L*.5:D← filtered dataset of features *X* with corresponding labelled pixel from *Y*.6:Split *D* into training data $D_{T}$ and testing data $D_{E}$.7:Fit an XGBoost classifier *C* using $D_{T}$.8:R← List of unique relevance values of processed features *X* from *C*.9:**for all** values **in**
*R*
**do**10: $D_{TF}\leftarrow$ Filtered underscored features from $D_{T}$.11: Fit *C* using $D_{TF}$.12: Append accuracy values from *C* into *T*.13:**end for**14:Fit *C* using the best features threshold from *T*.15:Validate *C* with k-fold cross-validation from $D_{TF}.$   ▹number of folds = 10
***Prediction***16:P← Predicted values for each sample in *X*.17:Convert *P* array into a 2D orthorectified image.18:O← Displayed/overlayed image.19:**return**
*O*

Reflectance cube bands are loaded into the program to calculate suitable spectral indexes and improve the detection rates as mentioned in Step 2. For this approach, the most traditional indexes, such as the normalised difference vegetation index (NDVI) [63], the green normalised difference vegetation index (GNDVI) [64], the soil-adjusted vegetation index (SAVI) [65], and the second modified adjusted vegetation index (MSAVI2) [66], are calculated. Additionally, two-dimensional (2D) smoothing kernels are applied into such indexes, following Equation (1).

(1) $$K=\frac{1}{w^{2}}\left[\begin{array}{cccc} 1 & 1 & \cdots & 1\\ 1 & 1 & \cdots & 1\\ \vdots & \vdots & \ddots & \vdots \\ 1 & 1 & \cdots & 1 \end{array}\right]$$

where *K* is the kernel of the filter, and *w* is the size of the window. Here, up to three kernels for w=3, w=7, and w=15 are calculated per vegetation index. All the hypercube bands in reflectance, as well as calculated vegetation spectral indexes, are denominated data features. In Step 3, an array of features is generated from all the retrieved bands *I* and the calculated indexes *S*.

### 2.3. Training and Prediction

The labelled regions from the ground-based assessments are exported from ArcMap and loaded into an array *Y*. In Step 5, an array *D* is created by filtering the features from *X* with their corresponding labels in *Y* only. Filtered data is separated into a training (80%) and testing (20%) data array. In Step 7, data is processed into a supervised XGBoost classifier. This model is utilised considering the moderate amount of labelled data (insufficient to run a deep learning model), the amount of information to be processed for a single hypercube, and the nature of the data from the exclusion trial (ground-based test). This classifier is currently a cutting-edge decision tree and gradient boosting model optimised for large tree structures, excellent performance, and fast execution speeds, outperforming detection rates of standard non-linear models such as random forests, k-NN, LDA, and SVM [59]. Moreover, input data features do not require any scaling (normalisation) in comparison with other models. Once the model is fitted, Step 8 retrieves the relevance of each processed feature (reflectance bands, spectral indexes, and transformed images) from the array *X* in a list *R*. The list is sorted to discard irrelevant features, increment the detection rates of the algorithm, avoid over-fitting, decrease the complexity of the model, and reduce computer processing loads.

Algorithm 1 executes a loop to evaluate the optimal number of ranked features that can offer the best balance between accuracy and data processing load. At each instance of the loop, specific features from the training set $D_{T}$ is filtered based on a threshold of relevance scores (Step 10). Later, the XGBoost model is fit using the filtered training set (Step 11) to record all accuracy values per combination. In Step 14, the best combination of accuracy and number of features is retrieved to re-fit the classifier. Finally, the fitted model is validated using k-fold cross-validation (Step 15).

In the prediction stage, unlabelled pixels are processed in the optimised classifier, their values displayed in the same 2D spatial image from the orthorectified hyperspectral cube. Ultimately, classified pixels are depicted using distinguishable colours and exported in tagged image file (TIF) format, a compatible file with georeferencing-based software.

## 3. Experimentation Setup

### 3.1. Site

**Figure 4:**
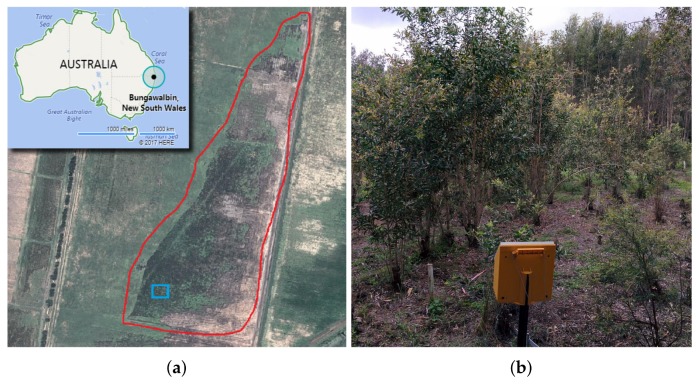
Site of the case study. (**a**) Location and covered area of the farm in red and experiment trees in blue; (**b**) Overview of paperbark tea trees under examination.

As displayed in Figure 4a, the experimentation occurred in a paperbark tea tree forest located near 71 Boggy Creek Rd, Bungawalbin, NSW 2469, Australia (29°04′42.9″ S 153°15′10.0″ E). Data acquisition was conducted on the 25 August 2016 at 11:40 a.m. Weather Observations for that day stated conditions of a partially cloudy day, with a mean temperature of 18.8 °C, a relative humidity of 46%, west-northwest winds of 20 km/h, a pressure of 1009.5 hPa, and no precipitation [67]. As seen in Figure 4b, the site includes selected paperbark trees that are monitored to assess the effects of myrtle rust on them.

### 3.2. Flight Campaign

The acquired data from flight campaign incorporated a single mission route. The UAV was operated with a constant flight height of 20 m above the ground, an overlap of 80%, a side lap of 50%, a mean velocity of 4.52 km/h, and a total distance of 1.43 km. The acquired hyperspectral dataset had a vertical and horizontal ground sample distances (GSD) of 4.7 cm/pixel.

### 3.3. Field Assessments

**Figure 5:**
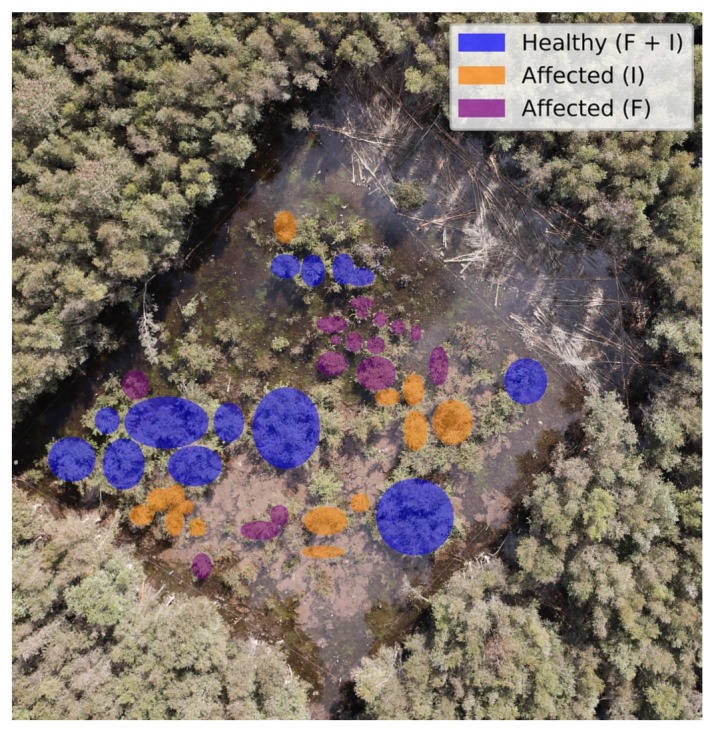
Aerial view of individual trees through on-ground assessments.

In order to evaluate the effects and flora response of myrtle rust on paperbark trees, a biological study in an exclusion trial on the mentioned site was conducted. Several on-ground assessments were conducted in individual trees within a replicated block design using four treatments: trees treated with fungicides (F), insecticides (I), fungicides and insecticides (F + I), and trees without any treatment action. Figure 5 illustrates the treatment methods the studied trees received.

From the indicators generated, insect and disease assessments were extracted to label every tree. Overall, the assessment report showed that only the trees that received insecticide and fungicide treatments remained healthy under direct exposure to the rust. In contrast, trees treated with insecticide were affected by rust and those treated with fungicide were affected by insects. Thus, trees treated with fungicides and insecticides were consequently labelled as healthy and the others as affected in the database.

### 3.4. Preprocessing

**Figure 6:**
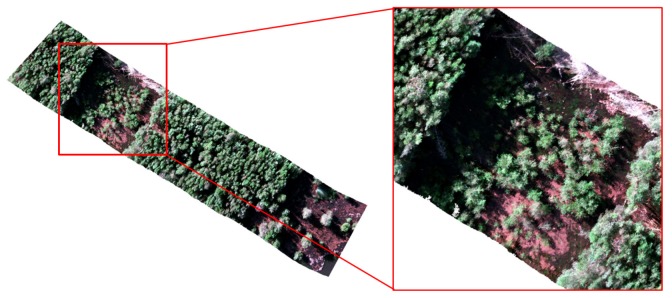
Red–green–blue (RGB) colour model representation of the orthorectified cube with its extracted region of interest.

As mentioned in Section 2.2, the entire surveyed area included an exclusion trial. As a result, an orthorectified hyperspectral cube in reflectance with spatial dimensions of 2652 × 1882 pixel of a 5.4 GB size was generated. This area was cropped from the original hypercube, reducing computational costs and discarding irrelevant data. Eventually, a 1.7 GB cube of 1200 × 1400 pixel as depicted in Figure 6 was extracted.

### 3.5. Training and Prediction

The XGBoost classifier contains several hyper-parameters to be set. Following a grid search technique, in which the classifier performance and detection rates were tracked with a set of possible hyper-parameters, the optimal values found for this case study were

estimators=100,learningrate=0.1,maximumdepth=3

where “estimators” is the number of trees, “learning rate” is the step size of each boosting step, and “maximum depth” is the maximum depth per tree that defines the complexity of the model.

## 4. Results and Discussion

**Figure 7:**
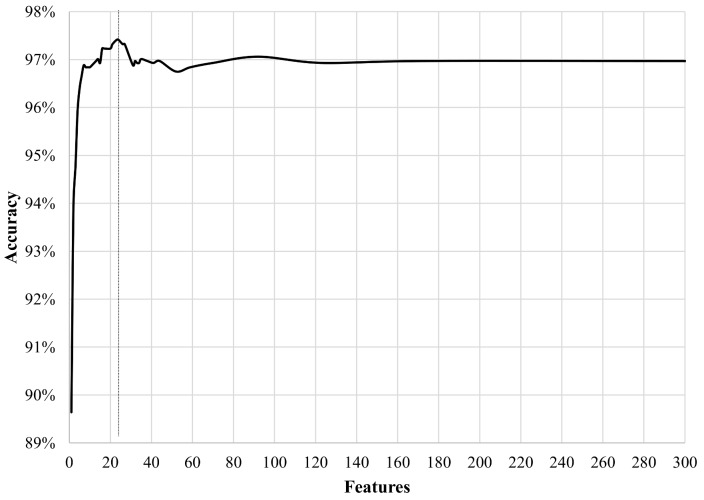
Performance of the classifier using different filtered features. Optimal number of features: 24.

To visualise the benefits of inserting an optimisation scheme in Step 8 of Algorithm 1, detection rates were tracked by training and running the classifier multiple times with only a set of filtered features per instance. The features were ranked with their relevance by the XGBoost classifier and sorted consequently, as illustrated in Figure 7.

**Figure 8:**
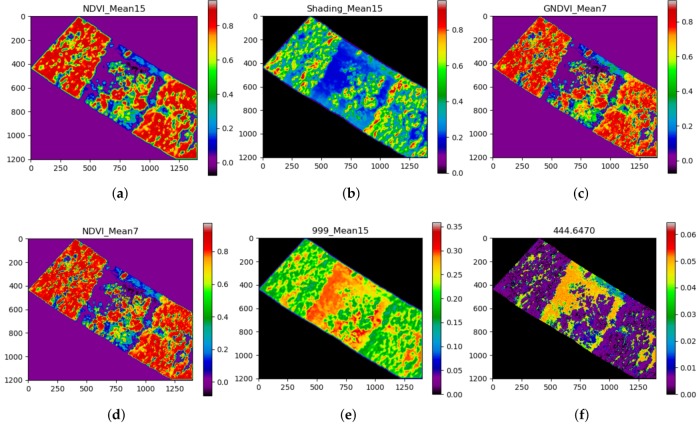
False colour representation of the first six features by relevance. (**a**) Smoothed NDVI with *k* = 15. (**b**) Smoothed Shading with *k* = 15. (**c**) Smoothed GNDVI with *k* = 7. (**d**) Smoothed NDVI with *k* = 7. (**e**) Smoothed 999 nm reflectance band with *k* = 15. (**f**) Raw 444 nm reflectance band.

The classifier can achieve high accuracy rates exceeding 97% of global accuracy when it processes data using from 10 to 40 features only, with an optimal number of features of 24. On the other hand, the classifier merely improves their registers when the number of processed features is more substantial. With this capability, the proposed approach can process fewer data and reduce the number of calculations to achieve high detection values. Additionally, this boosts the capability of the algorithm of processing large datasets in less time, an ideal scenario for mapping vast rural areas. The most relevant features of this study case are depicted in Table 1 and Figure 8.

It is shown how the first four features for this classification task come from specific vegetation indexes and processed images by 2D kernels—specifically, NDVI, shading, and GNDVI features (Figure 8a–d). Although their illustrations show insights of distinguishable intensities between healthy and affected tree regions from Figure 5, these sets of features are insufficient for segmenting areas of other objects. Thus, specific reflectance wavelengths bands such as 999 and 444 nm (Figure 8e,f) are also determinant. Additionally, features processed with 2D kernels obtained better relevance scores than their unprocessed counterparts. That difference was even greater for processed features using big window kernels considering that high amounts of noise, common in raw hyperspectral imagery, altered the performance of the approach. Nonetheless, these rankings do not suggest that these features can be used as global indicators to detect and map similar case studies (myrtle rust); the feature ranking table showed here is relevant to the fitted XGBoost model only, and results may differ if the same features are processed through other machine learning techniques. It is recommended, therefore, to perform individual analyses for every case study.

A total of 11,385 pixel contained in 23 features filtered by their relevance were read again in Step 14 of Algorithm 1. Data was divided into a training array $D_{E}$ with 9108 pixel and a testing array $D_{T}$ with 2277 pixel. The generated confusion matrix of the classifier and its performance report is shown in Table 2 and Table 3.

In sum, most of the classes were predicted favourably. The majority of misclassifications between the “Healthy” and “Affected” classes are possibly caused by human errors while labelling the regions manually in the raw imagery. Considering a weighed importance of precision and recall of 1:1, the F-support scores highlight a detection rate of 97.24% for healthy trees, 94.25% for affected trees, and an overall detection rate of 97.35%. Validation through k-fold cross-validation shows that the presented approach has an accuracy of 96.79%, with a standard deviation of 0.567%.

The performance of Algorithm 1 was tested in a computer with the following characteristics: Processor Intel${}^{®}$ Core™ i7-4770, 256 GB SSD, 16 GB RAM, Windows 7 64bit, and AMD Radeon™ HD 8490. It contains a report of the elapsed seconds for the application to accomplish the primary data processing, training, and prediction tasks, as illustrated in Table 4.

Taking into account the dimensions of the processed hypercube (1400 × 1200) and the initial number of bands (274), it was observed how a great demand of resources was required to open the file itself and calculate spectral indexes, accumulating 61.7 s on average. Similarly, the features filtering process in the training section also demanded considerable time, exceeding 50 s. On the other hand, the elapsed time executing the remaining tasks of the training phase was remarkably short. Specifically, the report highlights the benefits of filtering irrelevant features by comparing the duration of fitting the classifier for the first time with the duration of re-fitting it again with less yet relevant data from 8.74 to 0.99 s. Overall, the application spent 2 min and 51 s to evaluate and map an area of 338 m${}^{2}$ approximately.

**Figure 9:**
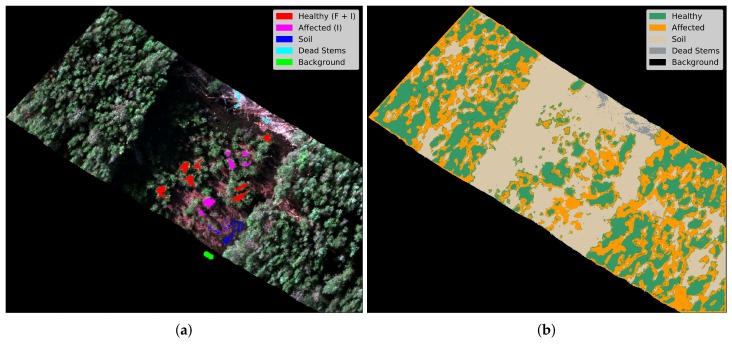
Segmentation results of the proposed approach. (**a**) Recovered hyperspectral image in red–green–blue (RGB) colour model; (**b**) Segmentation results.

**Figure 10:**
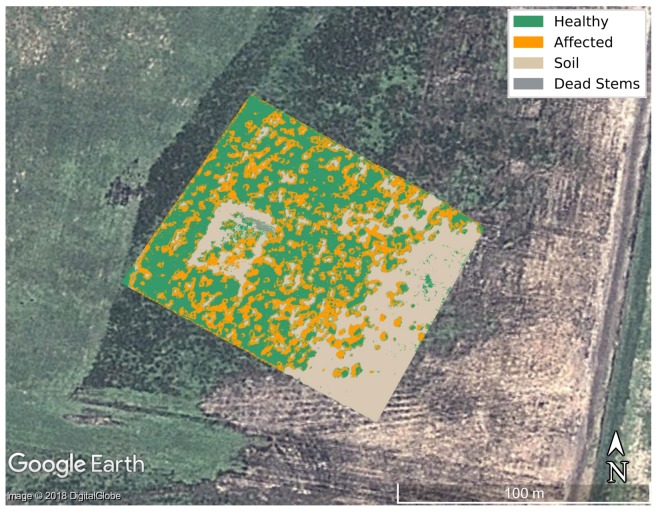
Layer of mapping results of the study area in Google Earth.

The GSD value of 4.7 cm/pixel from the acquired hyperspectral imagery represented a minor challenge in labelling individual trees, but is still problematic when specific stems or leaflets need to be highlighted. Higher resolution can assist in higher classification rates. As an illustration, the final segmented image of the optimised classifier is shown in Figure 9, where Figure 9a shows the digital labelling of every class region and Figure 9b depicts the generated segmentation output by Algorithm 1. A hypercube covering the entire area flown was also processed using the trained model, with results shown in Figure 10.

Results show a segmentation output using XGBoost as the supervised machine learning classifier that works well for this task. This classifier as well as Algorithm 1 are not only important for their capabilities to offer a pixel-wise classification task, but they also allow a rapid convergence, do not involve many complex mathematical calculations, and filter irrelevant data, compared to other methods. Nevertheless, it is suggested that their prediction performance be revised with new data. Like any model based on decision trees, over-fitting may occur, and misleading results might be generated. In those situations, labelling misclassified data, aggregating them into the features database and rerunning the algorithm is suggested.

The availability to process and classify data with small GSD values demonstrates the potential of UASs for remote sensing equipment compared with satellite and manned aircraft for forest health assessments on forest and tree plantations and with traditional estimation methods, such as statistical regression models. In comparison with similar approaches of non-invasive assessment techniques using UAVs and spectral sensors, this framework does not provide general spectral indexes that can be applied with different classifiers and similar evaluations. In contrast, this presented method boosts the efficiency of the classifier by receiving feedback from the accuracy scores of every feature and transforming the input data in consequence. The more explicit the data for the classifier is, the better the classification rates are. Furthermore, it is also demonstrated that a classifier which processes and combines data from multiple spectral indexes provides better performance than analysing individual components from different source sensors.

## 5. Conclusions

This paper describes a pipeline methodology for effective detection and mapping of indicators of poor health in forest and plantation trees integrating UAS technology and artificial intelligence approaches. The techniques were illustrated with an accurate classification and segmentation task of paperbark tea trees deteriorated by myrtle rust from an exclusion trial in NSW, Australia. Here, the system achieved detection rates of 97.24% for healthy trees and 94.72% for affected trees. The algorithm obtained a multiclass detection rate of 97.35%. Data labelling is a task that demands many resources from both site-based and remote sensing methods, and, due to human error, affects the accuracy and reliability of the classifier results.

The approach can be used to train various datasets from different sensors to improve detection rates that single solutions offer as well as the capability of processing large datasets using freeware software. The case study demonstrates an effective approach that allows for rapid and accurate indicators, and for alterations of exposed areas at early stages. However, understanding disease epidemiology and interactions between pathogens and hosts is still required for the effective use of these technologies.

Future research should discuss the potential of monitoring the evolution of affected species through time, the prediction of expansion directions and rates of the disease, and how data will contribute to improving control actions to deter their presence in unaffected areas. Technologically, future works should analyse and compare the efficacy of unsupervised algorithms to label vegetation items accurately, integrate the best approaches in the proposed pipeline, and evaluate regression models that predict data based on other biophysical information offered by site-based methods.

## Figures and Tables

**Table 1:** Ranking of the most 30 relevant features.

#	Feature	Score	#	Feature	Score	#	Feature	Score
1	NDVI_Mean15	0.0933	11	NDVI_Mean3	0.0247	21	975.3710	0.0119
2	Shading_Mean15	0.0780	12	759.9730	0.0212	22	671.1490	0.0109
3	GNDVI_Mean7	0.0563	13	999_Mean3	0.0212	23	893.2090	0.0109
4	NDVI_Mean7	0.0558	14	999_Mean7	0.0202	24	990.9150	0.0099
5	999_Mean15	0.0504	15	997.5770	0.0188	25	877.6650	0.0094
6	444.6470	0.0494	16	764.4140	0.0148	26	966.4890	0.0094
7	Specularity_Mean15	0.0380	17	444_Mean15	0.0143	27	766.6350	0.0084
8	999.7980	0.0341	18	462.4120	0.0133	28	853.2380	0.0079
9	GNDVI_Mean15	0.0286	19	NDVI	0.0133	29	935.4000	0.0079
10	444_Mean7	0.0267	20	Shading_Mean7	0.0133	30	GNDVI_Mean3	0.0079

**Table 2:** Confusion matrix of the eXtreme Gradient Boosting (XGBoost) classifier.

	Predicted	Healthy	Affected	Background	Soil	Stems
**Labelled**	Healthy	1049	15	0	0	0
	Affected	45	531	0	0	0
	Background	0	0	158	0	0
	Soil	0	0	0	321	0
	Stems	0	0	0	1	157

**Table 3:** Classification report of the confusion matrix of Table 2.

Class	Precision (%)	Recall (%)	F-Score (%)	Support
Healthy	95.89	98.59	97.24	1064
Affected	97.25	92.19	94.72	576
Background	100.00	100.00	100.00	158
Soil	99.69	100.00	99.68	321
Stems	100.00	99.37	99.68	158
Mean	97.32	97.32	97.35	∑ = 2277

**Table 4:** Performance in seconds of the main tasks from Algorithm 1.

Sub-Section	Instance 1	Instance 2	Instance 3	Instance 4	Instance 5	Mean	Std. Dev.
*Data preparation*							
Loading Hypercube	11.927	10.944	11.954	11.766	11.521	11.622	0.417
Calculating indexes	46.864	51.860	51.901	52.622	47.322	50.114	2.779
*Training*							
Preprocessing	0.152	0.141	0.149	0.148	0.140	0.146	0.005
Fitting XGBoost	8.948	8.654	8.758	8.679	8.692	8.746	0.119
Features Filtering	53.236	55.433	60.364	57.253	53.446	55.946	2.962
Re-Fitting XGBoost	0.964	1.023	1.010	0.998	0.965	0.992	0.026
*Prediction*							
Predicting results	29.738	40.749	42.131	34.473	66.477	42.714	14.188
Display	0.776	0.705	1.043	0.917	0.612	0.811	0.171
Total	152.607	169.508	177.309	166.857	189.175	171.091	13.489

## Abbreviations

The following abbreviations are used in this manuscript:

2D	Two-dimensional
3D	Three-dimensional
F	Fungicides
F + I	Fungicides and Insecticides
GDAL	Geospatial data abstraction library
GPS	Global positioning system
GNDVI	Green normalised difference vegetation index
GSD	Ground sampling distance
I	Insecticides
k-NN	k-nearest neighbours
LDA	Linear discriminant analysis
LiDAR	Light detection and ranging
MDPI	Multidisciplinary digital publishing institute
MSAVI2	Second modified soil-adjusted vegetation index
NDVI	Normalised difference vegetation index
NSW	New South Wales
RGB	Red–green–blue colour model
SAVI	Soil-adjusted Vegetation Index
SVM	Support Vector Machines
TIF	Tagged Image File
UAS	Unmanned Aerial System
UAV	Unmanned Aerial Vehicle
VNIR	Visible Near Infrared
WRIS	White Reference Illumination Spectrum
XGBoost	eXtreme Gradient Boosting
